# Forty Years Later—Complications and Mortality Associated with Cultured Epithelial Autografts in Burn Patients: A Systematic Review

**DOI:** 10.3390/bioengineering13101096

**Published:** 2026-09-22

**Authors:** Alexandre Dontschev, Vivienne Woodtli, Clemens Maria Schiestl, Kathrin Neuhaus, Philippe Abdel-Sayed, Anthony de Buys Roessingh, Christoph Moor, Ueli Moehrlen, Sophie Böttcher

**Affiliations:** 1 Pediatric Burn Center, Division of Plastic and Reconstructive Surgery, Children’s Skin Center, Department of Surgery, University Children’s Hospital Zurich, University of Zurich, Lenggstrassse 30, 8008 Zurich, Switzerland; vivienne.woodtli@kispi.uzh.ch (V.W.);; 2 Department of Pediatric Surgery, University Children’s Hospital Zurich, University of Zurich, Lenggstrasse 30, 8008 Zurich, Switzerland; 3 Children’s Research Center (CRC), University Children’s Hospital Zurich, University of Zurich, Lenggstrasse 30, 8008 Zurich, Switzerland; 4 Regenerative Therapy Unit, Service of Plastic, Reconstructive and Aesthetic Surgery and Hand Surgery, Lausanne University Hospital, University of Lausanne, 1011 Lausanne, Switzerland; 5 Children and Adolescent Surgery Service, Lausanne University Hospital, University of Lausanne, 1015 Lausanne, Switzerland; 6 Department of Pediatric Surgery, University Medical Center Mannheim, Medical Faculty Mannheim, Heidelberg University, 68167 Mannheim, Germany

**Keywords:** systematic review, CEA, burns, squamous cell carcinoma, mortality

## Abstract

Cultured epithelial autografts (CEAs) have been used for more than 40 years in the setting of extensive total body surface area (TBSA) burns. The development and clinical implementation of cultured epidermal autografts and, subsequently, combined cultured dermo-epidermal autografts (CDEAs) have provided an essential adjunctive treatment option. Over the past five decades, numerous studies have reported on their clinical application. CEAs are manufactured from autologous epidermal cells that are expanded in vitro and assembled into sheet grafts suitable for transplantation. While substantial literature exists regarding their clinical use, data specifically addressing treatment-related complications remain scarce. This systematic review aims to comprehensively synthesize the available evidence on sheet-type CEA/CDEA use in burn patients, with a particular focus on complications, tumorigenicity, and mortality. A systematic literature search was conducted in 2023 across five electronic databases (Cochrane on 31 May 2023, Embase on 31 May 2023, Ovid Medline on 31 May 2023, Scopus on 1 June 2023, and Web of Science on 1 June 2023) to identify studies reporting on the use of CEAs in burn patients. Search terms included combinations of “cultured,” “epithelial,” “autografts,” “take rate,” “mortality,” and “skin cancer.” Eligible studies were human clinical studies involving burn patients treated with sheet-type CEA or CDEA, published in English, French, or German. Exclusion criteria comprised studies involving allogeneic materials, in vitro or animal studies, and commentaries and editorials. Efficacy endpoints included complications, tumorigenicity, and mortality outcomes in CEA-treated patients. Risk of bias was assessed for each included study using the modified Downs and Black checklist and graded according to Hooper et al. Seventy studies contributing 72 patient cohorts published between 1984 and 2023 met inclusion criteria, encompassing a total of 2091 patients from all five continents. The mean graft take rate was 64% in adults and 70% in pediatric patients. Complication events and mortality data are described in the corresponding tables. This systematic review represents the first comprehensive synthesis specifically addressing complications, tumorigenicity, and mortality in burn patients treated with CEAs. The reported adverse events must be interpreted within the context of the high morbidity associated with extensive burn injuries. The observed mortality and number of complications appear primarily related to burn severity rather than to CEA use itself. No adverse events were identified that could be specifically attributed to CEAs. Interpretation of these findings is limited by the clinical and methodological heterogeneity of the included studies. In patients with extensive burn injuries, the use of CEAs is associated with a relatively low number of complications and appears to be a safe and clinically justified treatment option. This study received no internal or external funding. This study is referenced in the PROSPERO Database for systematic reviews under the number CRD420251040971.

## 1. Introduction

Despite modern advances, burn injuries remain a major challenge in both acute resuscitation and acute wound management. Major burns, involving an extensive body surface area (BSA), are associated with substantial morbidity, prolonged hospitalization, and increased mortality [1]. In these cases, early excision and wound coverage is a core therapeutic objective to mitigate massive release of inflammatory markers of the burned tissue, sepsis, and downstream complications [2]. Autologous split-thickness skin grafting (STSG) remains the standard of care; however, in major burns, donor-site availability can become a critical constraint, prompting the use of adjunctive or alternative coverage strategies.

Among these, cultured epithelial autografts (CEAs), first described by Rheinwald and Green in 1975 [3], enabled expansion of autologous keratinocytes from a small biopsy into epithelial sheets suitable for grafting. Cultured dermo-epidermal autografts (CDEAs) represent related approaches incorporating fibroblasts. Over the subsequent decades, CEAs/CDEAs have been adopted internationally, particularly in major burns, where conventional autografting is limited. Nevertheless, the literature describing outcomes has remained heterogeneous, with variation in indications, manufacturing pathways, application techniques, and postoperative wound care. Importantly, while case series frequently report graft take and short-term endpoints, a systematic synthesis focused specifically on complications, tumorigenicity, and mortality has been lacking.

Accordingly, this systematic review was designed to synthesize published evidence on CEA/CDEA use in burn patients between their first clinical use in the context of burns in 1984 up until 2023, with a primary focus on complications, tumorigenicity and mortality, and secondary extraction of treatment practices and core outcomes (e.g., graft take), to clarify the safety profile of CEA/CDEA in major burn care.

## 2. Materials and Methods

Five electronic databases were manually searched without automation tools, from inception to 1 June 2023: Cochrane (31 May 2023), Embase (31 May 2023), OVID MEDLINE (31 May 2023), Scopus (1 June 2023), and Web of Science (1 June 2023). Searches combined terms related to cultured autologous skin and burn outcomes (e.g., cultured, epithelial, autograft, autologous, tissue engineered, skin, burn, mortality, carcinoma, infection, contracture, and length of stay) using Boolean operators. In Embase, animal-only records were excluded using NOT ([animals]/lim NOT [humans]/lim).

Included studies were original clinical reports involving human burn patients treated with CEA and/or CDEA, with full text available in English, French, or German. Exclusion criteria were in vitro or animal studies; allogeneic skin substitutes without an autologous cultured component; editorials/commentaries/guidelines/conference abstracts; and studies lacking accessible full text. Studies were additionally excluded if they did not concern clinical grafting of CEA/CDEA sheets (e.g., non-sheet applications) or if the dataset was clearly duplicated within another included study.

Search results were imported into Zotero (v6.0.27, Falls Church, VA, USA) for deduplication. Title/abstract screening was performed manually by the first author, Alexandre Dontschev (AD). Records with uncertain eligibility were reviewed with a second reviewer, Clemens Schiestl (CS), and differences in opinion resolved based on the clinical and research experience of CS. No automation tools were used. The selection process was documented using a PRISMA 2020 flow diagram [4] (Figure 1).

Data were extracted for study characteristics (country, year, single- vs. multicenter, design, and prospective vs. retrospective), intervention characteristics (CEA vs. CDEA), and patient characteristics (sample size, age, and TBSA). Outcomes extracted included graft take (as reported), complications (overall and by category when available), and mortality. Mortality was additionally tabulated by 10% TBSA strata (e.g., 1–9%, 10–19%, etc.) when extractable from the original report. Mean age, TBSA, and take rate were calculated by multiplying the average item by the number of patients in one study, repeating this process for all the included studies and summing them up together, and then dividing this sum by the total amount of patients. Take rate is defined as the quotient of the grafted area divided by the area surviving graft after a defined period has passed. Complications have been reported as separate outcome than mortality and consequently the complications have been reported as events and not as patients, since more than one complication can happen in one patient. Levels of evidence were assigned according to the Oxford Centre for Evidence-Based Medicine 2011 framework [5].

Missing or unclear data were defined as absence of one or more of the following: (a) age information, allowing classification into adult/pediatric strata; (b) stratum-specific sample sizes; or (c) explicit reporting of a complication/death or explicit statement of no complications. Analyses were restricted to extractable, unambiguous data from the original publications. 

To assess risk of bias, the modified Downs and Black checklist [6] was used for every study, giving a numerical score to each study. This assessment was done by AD, without automation tools. The studies are scored from 0 to 28, with 0 being the most at risk for bias, and 28 the least at risk for bias. The studies were given a grade according to Hooper et al. [7], ranging from excellent (least risk of bias) if the study scores between 26 and 28 to poor (most at risk of bias) if the study scores < 15. To calculate this score, each study must be graded according to a series of 27 questions that assess different sources of potential bias.

A formal meta-analysis could not be performed due to substantial heterogeneity across the included studies and the lack of consistent control groups. The heterogeneity observed encompassed clinical, methodological, and statistical dimensions. Clinical heterogeneity was evident in the populations treated, indications, burn severity, surgical approaches, and grafting techniques. The included studies span nearly four decades of CEA use, during which clinical practice evolved considerably. Differences in culture techniques, wound-bed preparation, adjunctive treatments (e.g., split-thickness autografts), and supportive care make direct comparison of clinical outcomes difficult. These variations were reviewed qualitatively and judged by the authors to indicate meaningful clinical heterogeneity. Methodological heterogeneity was observed across study designs. Most studies were retrospective case series without control groups, and the reporting of key variables (e.g., TBSA, depth of burn, graft take, complications, and survival) was inconsistent. Some studies reported aggregate descriptive outcomes only, while others provided more detailed quantitative data. This variability limits the ability to extract standardized effect sizes or directly compare results across studies. Statistical heterogeneity could not be formally assessed. The absence of comparable outcome measures, absence of control groups, and frequent lack of variance estimates (e.g., standard deviations and confidence intervals) prevents the calculation of pooled effect estimates or heterogeneity statistics. As a result, quantitative synthesis was not feasible. Given the heterogeneity in design and reporting, data were synthesized descriptively. No meta-analysis was performed, due to heterogeneity and the lack of consistent control groups. No tools were used to explore the heterogeneity of the included studies. We conducted no sensitivity analysis. A formal statistical assessment of reporting bias was not feasible, due to the absence of a meta-analysis, the heterogeneity of study designs, and the lack of comparable quantitative outcome measures across studies. We performed no formalized certainty assessment. No protocol was prepared for this review. This study is referenced in the PROSPERO Database for systematic reviews under the number CRD420251040971.

## 3. Results

A total of 70 studies contributing 72 patient cohorts met inclusion criteria (Table 1). Included studies comprised 65 single-center and five multicenter reports. Designs included case series (40), case reports (18), case–control (eight), observational studies (three), and cohort studies (one). Fifty-nine studies were retrospective, and 11 prospective. Sixty-seven studies were published in English, and three in French; no German-language studies were identified despite eligibility (Figure 2). CDEA grafting concerned 10 patients out of the total included patients.

Across included reports, a total of 2098 patients were treated with CEA, 1294 adults and 427 pediatric patients. Pediatric patients were defined as being under the age of 16 years old. Missing data to permit an age classification into pediatric or adult groups concerned 377 patients; these patients were excluded from the subsequent analysis (Table 2. Patients were treated between 1984 and 2023. CDEA grafting concerned 10 patients (0.47%) out of the total included patients. The weighted mean TBSA burned was 64% in adults and 58% in pediatric patients, and the overall weighted mean was 62% (Table 3). Weighted mean graft take rate was 64% in adults and 70% in pediatric patients. Mean follow-up time was variable, with a mean of 22 months, with the longest follow-up being 10 years following CEA grafting to treat contractures following the initial operation [58].

Among the 2098 patients, data on complications and tumorigenicity (Table 4) were available for 953 adults and 383 pediatric patients, with missing data concerning 762 patients and therefore were excluded from complications calculation and analysis. Importantly, mortality was extracted as its own endpoint, separately to the complications, and was not counted toward a “complication” event. Complication data are described in Table 2.

Mortality data were extractable for 1275 patients. Overall, 174 deaths occurred, corresponding to an overall mortality of 13.6%, with a TBSA of 58%. Mortality was stratified by 10% TBSA categories (Table 5). Mortality was additionally stratified by age using a cutoff of 16 years: among adults, 149 deaths occurred in 942 patients (15.8%), and among pediatric patients, 25 deaths occurred in 333 patients (7.5%).

Risk of bias was assessed using the previously mentioned approach, 38 studies were classified as “fair” and 32 as “poor.”

## 4. Discussion

To our knowledge, this is the first systematic review to comprehensively evaluate the long-term safety and clinical outcomes of CEAs in the treatment of major burn injuries. By synthesizing more than four decades of published experience, we demonstrate that CEAs are associated with a favorable complication profile, no increased risk of long-term cutaneous malignancy and positive effect on survival in patients with extensive burns. Although the available evidence is largely derived from retrospective case series and heterogeneous study populations, the findings support the continued use of CEA as an important adjunct in the management of major burns. At the same time, the review highlights the need for standardized reporting and prospective long-term follow-up to better define the safety and effectiveness of this technology.

### 4.1. Complications

Despite the inability for us to perform a meta-analysis of the complication data, due to heterogeneity in reporting and definition of the different complications, we descriptively highlight a low number of complications. The event “no complications” represents 77.75% of all patients and local complications of the CEA, best described by combining the number of events in the categories of “contracture,” “graft loss,” and “bleeding/ulceration/blistering/hypopigmentation,” representing 16.3%, which remains an acceptable number of local complications, given the severity of the burns for which CEA is indicated. Regarding contractures, Alsaif et al. [78] have described contractures in 30% of hand-burn patients grafted with STSG in their systematic review. Aesthetic appearance and pigmentation have been described as satisfactory by Alsaif et al. [78], and CEA also has a favorable profile of cosmetic appearance, as described by Coleman et al. [24]. They have also described pigmentation being satisfactory to patients. Ünal et al. [79] report graft loss due to infection in a prospective study, yielding a 47.4% graft loss using STSG, contrasting the results of our systematic review regarding graft loss. Major complications are unlikely to be attributable solely to CEA use, as major burns are de facto more prone to complications [80], regardless of what surgical technique has been used to treat patients. In this regard, the number of complications described in the included patients should not deter from the use of CEAs in large burns.

### 4.2. Tumorigenicity

Malignant transformation of burn wounds is a documented phenomenon [81], based on case descriptions, first described over two centuries ago and classically conducive to Marjolin’s ulcer, a form of cutaneous squamous cell carcinoma (SCC) that arises in areas of prolonged inflammation, scarring, or non-healing wounds. Due to the culture technique of CEA, involving a feeding layer of irradiated 3T3 murine cells [3], exploring the link between application of CEA and tumorigenicity is of particular interest regarding the safety aspect of CEA. Furthermore, investigating this link is of importance, since squamous cell carcinoma arising from a burn injury tends to be more aggressive than sun-induced SCC [82,83]. The risk of malignant degeneration in chronic wounds has historically been estimated at approximately 1.8% [84], with a latency of over 30 years [81]. In this systematic review, a total of eight cases of SCC in three adult patients were identified among 2105 patients who received CEA transplantation, representing 0.3% of all patients. All reported cases occurred at least more than ten years after the initial grafting procedure [39,66,74]. Importantly, no other types of skin malignancies, including malignant melanoma (MM) or basal cell carcinoma (BCC), were reported in any of the included studies.

This observed incidence of SCC must be contextualized within broader epidemiological data. A Swedish cohort study [85] involving 37,095 burn patients reported no increase in skin cancer risk compared to the general population, with standardized incidence ratios (SIRs) of 0.88 for both SCC and MM (95% CI: 0.70–1.09 and 0.68–1.12, respectively). Similarly, a large Danish cohort study [86] found no elevated cancer risk in burn survivors. In that study, 18 SCCs, 21 melanomas, and 94 BCCs were observed over a 25-year period. The standardized incidence ratio (SIR) for SCC was 0.9 (95% CI: 0.6–1.5), indicating no significant deviation from the expected baseline. In fact, the risk for MM and BCC was reduced, with SIRs of 0.7 (95% CI: 0.4–1.1) and 0.7 (95% CI: 0.6–0.9), respectively. Additionally, depth of burn injury did not correlate with development of a specific type of skin cancer. Moreover, there was no higher risk of malignant skin cancer development between split-thickness skin-grafted patients and non-skin-grafted patients. The authors concluded that burn injuries do not appear to increase the overall risk for skin cancer.

While isolated cases of SCC have been documented in CEA-treated patients, the rarity, delayed onset, and absence of other skin-cancer types strongly suggest that these events are more likely attributable to the natural history of chronic wound biology in high-risk burned skin rather than to any direct oncogenic potential of the grafted material. Thus, current evidence supports the conclusion that CEA transplantation does not increase the risk of skin cancer.

### 4.3. Mortality and Survival

Beyond complications and tumorigenicity, if one considers mortality as the key endpoint in a complex therapy such as the treatment of burns, the following figures take on particular significance. The favorable overall take rate of 64% in adults and 70% in pediatric patients contributes greatly to reducing the mortality in large burns treated with CEAs, as the physiological barrier of the skin is restored. The analysis of mortality across the included studies revealed an overall 13.6% mortality among all patients treated with CEAs, where the raw mortality data were available. The subgroup of patients with a TBSA 70–79.9% included 226 patients and a mortality of 16.8%, which is in stark contrast to the figures reported by Walker et al. [87] in the United Kingdom (UK), showing a mortality rate of 71.9% in patients, with an average TBSA of 71.8% in a cohort of 96 patients with burn injuries with >50% TBSA treated using standard of care methods in a UK burn center. Since CEA tends to be used in the setting of major burns, our figure of 13.6% mortality represents an overall low percentage of deaths compared to the extent of the burn injury. This figure is also supported by the works of Matsumura et al. (2016) [52], which have shown a reduced mortality in a CEA-treated group, as compared to the standard of care. When compared to the mortality outcomes in each TBSA category from the American National Burn Repository [88], the mortality rate we describe appears comparatively favorable. Furthermore, in a Uruguayan retrospective cohort including 3132 patients between 1998 and 2018, the mortality rate for patients with a TBSA >40% was 75.2% [89]. However, such direct comparisons must be approached cautiously due to differing cohort characteristics, time frames, and supportive care practice. Nonetheless, our data point to the significant potential survival benefit of CEAs in the management of extensive burns.

This decrease in mortality of patients treated with CEAs reflects a global trend to reduce mortality since the 1970s. Since then, the lethal area 50 (LA50), the burned area at which, statistically, the mortality lies at 50%, has been decreasing, owing to the use of antibiotics, the centralization into highly specialized burn centers, and the increasing capabilities of bioengineered tissues. This last application has pushed the LA50 over 90% TBSA since 2005, as evidenced by Kasten et al. in 2011 [80]. In the same trend, the work of Garcia-Diaz et al. [90] shows that the mortality of burn injuries in Spain between 1978 and 2023 has decreased in part due to the advent of CEAs [90].

### 4.4. Regulatory Considerations

The regulatory environment ultimately dictates how and when CEAs are allowed to be used [91]. In this regard, the European Regulation No. 1394/2007 [92] in 2009 sets the standards necessary to produce and monitor the production of CEA and other skin derivatives. Based on this regulation, each national medicine agency can allow the clinical use of CEA. In this regard, Japan and the Republic of Korea hold a progressive approach to regenerative-medicine regulatory environments. Japan has had JACE^®^ (Japan Tissue Engineering Co., Ltd., Gamagori, Japan) on the market since 2007, via the use of an accelerated approval pathway destined to regenerative-medicine products, reflecting a systemic facilitator to the access of CEA for burned patients. The Republic of Korea has had full reimbursement by the national Industrial Accident Insurance for autologous cultured epidermal grafts since 2002. In the United States of America (USA), Epicel^®^ (Vericel Coproation, Cambridge, MA, USA) has been in use for humanitarian purposes since 1998; clinical approval in the USA, however, has existed since 2007, reflecting a lengthy process of evaluation for commercialization in the USA market [93].

The international regulatory environment, specific to each political entity, poses great challenges in a constantly evolving field with new techniques being developed at a rapid pace and may ultimately hinder the ability of eligible patient to receive CEA. This in turn reflects a possible selection bias in our study, because of the great number of different regulatory agencies, which can have different criteria to allow or disallow the use of CEA. In established countries, such as in Switzerland, the USA, or Japan, where the CEA production has been happening for decades, these administrative challenges have, to a large extent, been overcome. However, the regulatory environment is set to change according to the increasing development complexification and clinical use of CEA and skin tissue substitutes, as evidenced by the recent events in Crans-Montana following an indoor fire that injured more than a hundred people and left 40 people dead [94].

### 4.5. Limitations and Future Directions

The mortality statistics are also a source of bias, because of the associated publication bias. There is no formal way to access this gray data, where CEAs did not contribute to improved survival or, worse, contributed to higher mortality. The benefit of CEAs in burns patients is reflected in the mortality statistics, and it would seem unlikely that in the decades-long existence of CEAs, no publication exists showing an excess mortality compared to standard of care if it was indeed the case. Additionally, no standardized protocol for the preparation, application, or postoperative management of CEAs was identified, resulting in procedural heterogeneity across centers and time periods. Furthermore, the methodological quality of the included literature was low, with most studies falling into evidence level IV or V and relying on small, retrospective case series without control groups. Missing data affected one third of the included patients, limiting the possibility of a complete statistical analysis for some endpoints. Despite these limitations, this review represents the most systematic and comprehensive effort to date to synthesize the complications associated with CEA in the treatment of burn injuries, offering a broad and systematized overview of long-term safety outcomes in this patient population.

In this regard, a multicentric, observational long-term study of the outcomes of CEAs (Swisskera) is currently being set up in Switzerland to be able to characterize scar quality, incidence of skin cancer, and the need for reoperation due to contractures, in addition to other endpoints. Moreover, promising lower-cost methods of CEA production are being studied and clinically applied with promising results, as evidenced by Kleintjes et al. [95].

## 5. Conclusions

In conclusion, there is evidence that CEA has a low number of complications, does not lead to increased skin tumorigenicity, and has a low associated mortality. It represents a safe surgical solution in the setting of major burns, despite the manufacturing and regulatory challenges.

## Figures and Tables

**Figure 1:**
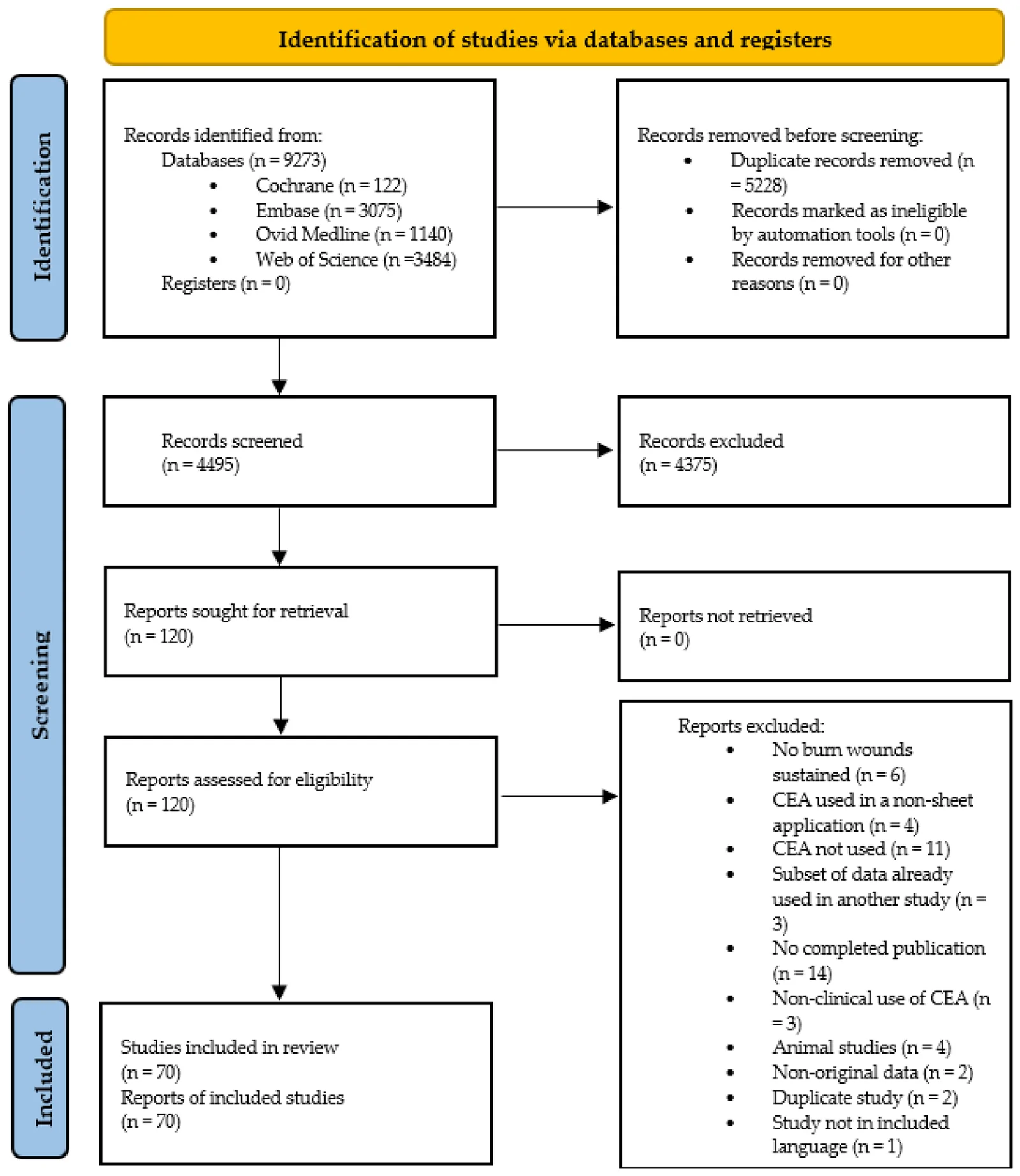
Flow diagram of included studies according to the PRISMA 2020 guidelines.

**Figure 2:**
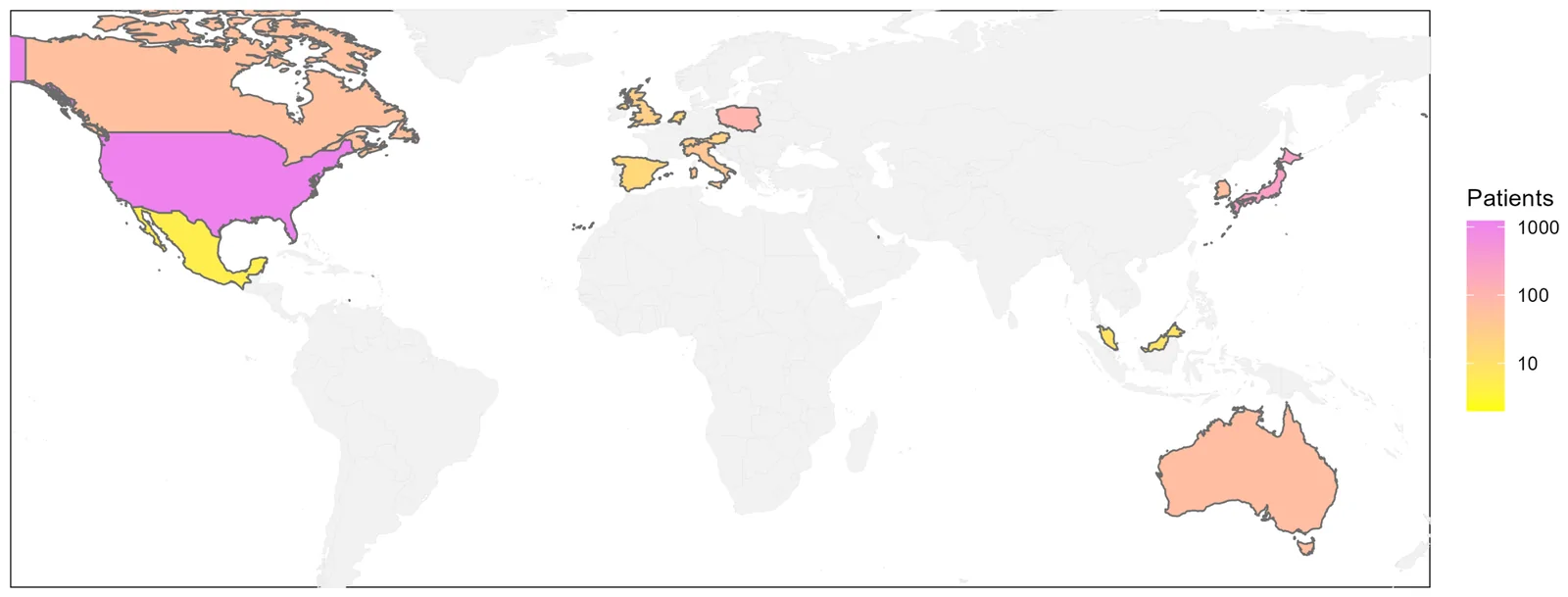
Geographic distribution of patients by country. This figure describes the number of patients per country that were included in our review.

**Table 1:** Included studies.

Reference	Author	Publication Year	Country	Study Type	Mean Age (Years)	*N* Patients	Mean TBSA (%)	Mean Take Rate (%)	Risk of Bias Score
[8]	Akita et al.	2018	Japan	Case series	52.4	8	44.87	86.25	18
[9]	Auxenfans et al.	2015	France	Case series	N/A	63	N/A	N/A	16
[10]	Ayala et al.	2022	USA	Case report	43.5	2	68	75	15
[11]	Barillo et al.	1992	USA	Case report	36.66	3	59	60.83	14
[12]	Barret et al.	2000	USA	Case–control	10.5	8	92.5	44	17
[13]	Bettex-Galland et al.	1988	CH	Case report	6	1	40	N/A	15
[14]	Blight et al.	1991	UK	Case series	N/A	26	50	15	14
[15]	Braye et al.	2001	France	Case series	35.28	14	69.28	67.57	15
[16]	Braye et al.	2000	France	Case series	10.6	12	60	84.16	15
[17]	Carsin et al.	2000	France	Case series	28.61	30	77.83	68.5	18
[18]	Caruso et al.	1999	USA	Case report	12	1	81	40	13
[19]	Chalumeau et al.	1999	France	Case series	7.5	6	82.33	84.33	15
[20]	Chrapusta et al.	2014	Poland	Case series	N/A	20	N/A	N/A	18
[21]	Chua et al.	2018	Singapore	Case series	33	10	52.5	N/A	17
[21]	Chua et al.	2018	Singapore	Case series	37.07	14	63.57	N/A	17
[22]	Cirodde et al.	2011	France	Case series	30	63	81	65	18
[23]	Clugston et al.	1991	Canada	Case–control	37.05	18	49.44	32.64	16
[24]	Coleman et al.	1992	USA	Case series	5.83	3	46.66	86.66	14
[25]	De Luca et al.	1989	Italy	Observational study	22.15	40	53.1	37.3	16
[26]	Doherty et al.	1986	USA	Case reports	6	2	97.5	52	11
[27]	Dorai et al.	2008	Malaysia	Case series	10.4	9	35.1	N/A	11
[28]	Efanov et al.	2018	Canada	Case series	40	9	70.33	N/A	16
[29]	Eliott et al.	2002	Australia	Case reports	33	2	75	N/A	14
[30]	Foyatier et al.	1990	France	Case series	34	16	66	N/A	14
[31]	Gallico et al.	1984	USA	Case report	5.5	2	97.5	N/A	15
[32]	Gobet et al.	1997	CH	Case series	8.16	6	57.16	65.83	16
[33]	Haith et al.	1992	USA	Case report	39.16	6	55.5	78.2	16
[34]	Hayashi et al.	2014	Japan	Case series	56.38	8	51.63	80	18
[35]	Hayashi et al.	2018	Japan	Case series	57.33	3	50.16	95	15
[36]	Herndon et al.	1992	USA	Case series	N/A	47	N/A	N/A	15
[37]	Herzog et al.	1998	USA	Case series	16.22	8	49.875	51.25	16
[38]	Hickerson et al.	1994	USA	Case series	37.6	5	59.6	93.6	15
[39]	Hickerson et al.	2019	USA	Case series	39.8	629	67.3	67.1	17
[39]	Hickerson et al.	2019	USA	Case series	N/A	206	N/A	N/A	17
[40]	Kan et al.	2022	Japan	Case report	7	1	80	N/A	13
[41]	Khalfan et al.	1989	Bahrain	Case report	53.5	2	22.5	90	12
[42]	Kitala et al.	2019	Poland	Case–control	41	81	55	N/A	17
[43]	Krupp et al.	1992	CH	Case series	N/A	21	N/A	55.62	11
[44]	Kumagai et al.	2002	Japan	Case series	N/A	34	N/A	73	11
[45]	Kym et al.	2015	Republic of Korea	Observational study	41.2	61	66.5	N/A	19
[46]	Latarjet et al.	1987	France	Case report	21.5	2	42.5	N/A	10
[47]	Llames et al.	2006	Spain	Case series	28.38	13	N/A	56.92	12
[48]	Lo et al.	2019	Australia	Observational study	49	10	55	40	15
[49]	Lopez Gutierrez et al.	1995	Spain	Case series	3.25	4	80.5	N/A	13
[50]	Loss et al.	2000	CH	Case report	26	1	93	N/A	11
[51]	Matsumura et al.	2016	Japan	Case series	20.1	13	80	38	16
[52]	Matsumura et al.	2016	Japan	Cohort study	49	216	50	66	12
[53]	McAree et al.	1993	USA	Case series	21	7	65.71	79.57	15
[54]	McGovern et al.	2023	USA	Case series	23.66	3	71.66	86.66	15
[55]	Moghtader et al.	1994	USA	Case report	37	1	35	100	11
[56]	Munster	1996	USA	Case–control	31.8	22	71.8	N/A	16
[57]	Nuñez-Gutiérrez et al.	1996	Mexico	Case–control	24.6	5	53	77.5	15
[58]	Nuri et al.	2018	Japan	Case report	N/A	1	20	N/A	12
[59]	Odessey	1992	USA	Case series	28	104	50	60	14
[60]	Paddle-Ledinek et al.	1997	Australia	Case series	21.3	37	65.64	58.32	14
[61]	Rab et al.	2005	Austria	Case–control	1.94	22	18.5	100	19
[62]	Rives et al.	1995	France	Case series	11.37	4	85.75	N/A	13
[63]	Rue et al.	1993	USA	Case series	29.75	16	63.65	49.8	17
[64]	Sheridan et al.	2001	USA	Case–control	6.4	7	75.9	45.7	17
[65]	Sheridan et al.	1995	USA	Case series	11.41	5	94	51	15
[66]	Singh et al.	2015	USA	Case report	N/A	1	95	N/A	11
[67]	Siwy et al.	1992	USA	Case series	31.6	5	65	75.3	12
[68]	Soeda et al.	1993	Japan	Case series	5.4	6	78.33	72.5	12
[69]	Sood et al.	2009	USA	Case series	4.27	19	51.875	75.21	13
[70]	Still et al.	1996	USA	Case report	14	1	58	100	13
[71]	Still et al.	1994	USA	Case series	N/A	15	N/A	66.62	12
[72]	Takami et al.	2014	Japan	Case series	42.25	4	64.75	94.75	12
[73]	Teepe et al.	1990	Netherlands	Case series	31.82	17	56.11	27.03	13
[74]	Theopold et al.	2004	USA	Case report	34	1	95	N/A	13
[75]	Thompson et al.	1987	Australia	Case report	41	1	55	N/A	13
[76]	Williamson et al.	1995	Canada	Case–control	35.3	28	52.2	26.09	16
[77]	Wood et al.	2006	Australia	Case series	N/A	14	60.7	N/A	15

Refs. [21,39] both contribute 2 patient cohorts in one publication. Mean age is calculated in years; mean TBSA and take rate are calculated in percentages. N/A = missing data.

**Table 2:** Available and missing data.

	Available Data	Adult	Pediatric	Missing Data
Age	1721	1294	427	377
TBSA	1692	N/A	N/A	406
Take rate	1455	N/A	N/A	593
Complications	1336	953	383	762
Mortality	1275	942	333	823

Number of patients with available data on age, TBSA, Take rate, complication, and mortality. N/A = missing data.

**Table 3:** TBSA data.

TBSA [%]	*N*	Adult	Pediatric
0–9	1	1	0
10–19	11	6	5
20–29	24	12	12
30–39	62	41	21
40–49	141	108	33
50–59	183	132	51
60–69	230	169	61
70–79	228	174	54
80–89	244	196	48
90–99	151	103	48

**Table 4:** Complication data.

Complications (Events)	*N*	Adults	Pediatric
No complications	741	626	115
Contracture	62	56	6
Graft infection/loss	59	56	3
Bleeding/ulceration/blistering/hypopigmentation	35	25	10
Squamous cell carcinoma (SCC)	8	8	0
Major complications	161	161	0

*N* = Total number of patients. Complication data are listed as events, as more than one complication can occur in one patient. Eight total cases of SCC were reported in 3 patients on CEA grafted sites.

**Table 5:** Mortality data per TBSA category.

Mortality per TBSA [%]	*N*	Adult	Pediatric
0–9	0	0	0
10–19	6	1	5
20–29	0	0	0
30–39	3	3	0
40–49	14	12	2
50–59	13	12	1
60–69	20	18	2
70–79	36	29	7
80–89	38	34	4
90–99	49	40	9
Total	174	149	25

## Data Availability

The original contributions presented in this study are included in the articles cited in the “References.” Further inquiries can be directed at the corresponding authors.

## Abbreviations

The following abbreviations are used in this manuscript:

CEA	Cultured epithelial autograft
TBSA	Total Burn Surface Area
CDEA	Cultured dermo-epidermal autograft
STSG	Split-thickness skin grafting
PRISMA	Preferred Reporting Items for Systematic Reviews and Meta-Analyses
OCEBM	Oxford Center for Evidence-Based Medicine
ID	Identification
SCC	Squamous cell carcinoma
MM	Malignant melanoma
BCC	Basal cell carcinoma
SIR	Standardized incidence ratio
UK	United Kingdom
LA50	Lethal area 50
USA	United States of America
